# Interaction between Age, Sex, and Mental Health Status as Precipitating Factors for Symptom Presentation in Concussed Individuals

**DOI:** 10.1155/2019/9207903

**Published:** 2019-12-31

**Authors:** Katie Lariviere, Samantha Bureau, Cameron Marshall, Matthew R. Holahan

**Affiliations:** ^1^Department of Neuroscience, Carleton University, Ottawa K1S 5B6, Canada; ^2^Complete Concussion Management, Inc., Toronto L6H 7W1, Canada

## Abstract

Concussions are among the most common neurological conditions, with emergency departments and sports injury clinics seeing hundreds of patients each year. The consideration of risk factors such as age, sex, and comorbid conditions are very important when looking at individual physiological and psychological outcomes after a concussion. The purpose of this study was to look at four comorbid conditions (depression, anxiety, behavioural disorder, or learning disability) and identify any interactions with age and sex in symptom presentation after suffering a concussion. A total of 4,865 participants from the CCMI (Complete Concussion Management Inc.) dataset were used with 1,577 self-identified with a diagnosis of anxiety, depression, a behavioural disorder, or a learning disability. Fixed-factor analyses of variance were used with age and sex as fixed, grouping factors and symptom total and severity as dependent measures. For the individuals who did not have one of the 4 mental health conditions (3,288 control participants), symptom total and symptom severity increased with age (*p* < 0.05), and females showed more symptoms and a higher symptom severity than males across all ages (*p* < 0.05). A diagnosis of anxiety or depression exacerbated total symptoms and symptom severity from 25–50% above control levels in the 19 and under age groups, while depression or anxiety exacerbated total symptoms and severity by 10–15% in males more than females over 20. A diagnosis of a behavioural disorder or a learning disability exacerbated symptom severity by approximately 50% above control levels in 13–19–year-old females and in males of 30 years and older. This study highlights how the presence of a mental health condition may alter concussion symptom presentation dependent on age and sex. The identification of risk factors and how they may interact can be of great value to health care providers who manage concussion symptoms and recovery.

## 1. Introduction

Mild traumatic brain injuries (mTBI) are among the most common neurological conditions, affecting millions of people each year [1]. A concussion is often described as a subset of mTBI, whereby there are rapid changes in neurological function that are short-lived and resolve spontaneously [2–4]. In teenagers and young adults between the ages of 15 and24, sport-related concussions (SRCs) are second to motor vehicle accidents [5]. In sex-comparable sports, the data also reveal higher rates of concussions in females than males [6]. Headache and dizziness are the most common concussion symptoms, with some of the other well-known symptoms being confusion, light sensitivity, nausea, fatigue, and amnesia [7]. Loss of consciousness may also occur, but it is seen in less than 5–10% of patients who suffer a concussion [7]. Prolonged loss of consciousness indicates a more severe brain injury [5]. In some cases, symptoms may not surface until days or weeks following the injury and can even be missed or misdiagnosed at first because the individual may appear to be fine. In addition, the under-reporting of concussions and concussion-like symptoms continues to be a serious medical concern, especially with athletes [8]. Those who continue to participate in sports after failing to report symptoms of a suspected concussion remain vulnerable to worsened symptomology if they return to play prior to full recovery [9].

Understanding how age, gender, and certain comorbid conditions affect the symptomology of a concussion is an essential aspect of concussion management [10]. The idea that sex plays a role in symptom presentation and recovery after a concussion is a consistent pattern in the literature [6]. In one study, females self-reported significantly more symptoms following a concussion than males [11]. In that study, females showed a greater degree of impaired cognitive performance compared with males [11]. Females were found to be 2 times more likely than males to report severe noise sensitivity following a concussion [12]. Males, on the contrary, show a higher incidence of anterograde amnesia and loss of consciousness than females [13]. The duration of symptoms shows sex differences with a median of 11 days for males to recover compared with the 28 days for females [13]. This study also reported that 75% of male athletes showed full recovery from their concussion by 3 weeks compared with 42% of females [13].

Age also plays a significant role in concussion symptom presentation with peaks occurring in young adulthood (15–19 years old) and those over 65 years old [12]. Among individuals older than 65, falls are responsible for more than 50% of concussions, while in young adults, motor vehicle accidents and sports-related concussions are most common [12].

Another factor that may contribute to symptom presentation following a concussion is the presence of a pre-existing mental health condition [14]. Of the studies that have examined this association, many have shown that a history of mental health issues is associated with worse symptom presentation after suffering a concussion [15]. Student-athletes with a diagnosis of anxiety, depression, or anxiety with depression reported more total symptoms and greater symptom severity than controls [16, 17]. Depression has also been suggested to be a predominant factor in prolonged recovery times and longer duration of postconcussive syndrome [18].

Children with neurodevelopmental disorders such as ADHD have been found to have a greater lifetime history of concussion [19, 20] but this is not a consistent finding [15]. Those with ADHD have different baseline scores during cognitive testing, which could contribute to the differences observed in symptomology [15, 20]. Cross-sectional studies have shown that the rate of concussions is elevated in youth with a diagnosis of ADHD, but these studies were not able to determine if ADHD was a risk factor for concussions or vice versa [21]. Another study showed that there was a significantly earlier age of onset of ADHD symptoms compared with the age of the concussion occurrence, and pre-existing ADHD contributes to longer recovery times [22]. This suggests that ADHD is an antecedent risk factor for concussion, and its presence may complicate the recovery course [22].

Those with learning disabilities have also been found to have a greater lifetime history of concussion [15]. Results showed that athletes with learning disabilities obtained lower baseline neurocognitive scores, which could have an impact on symptom reporting and symptom baseline scores [23]. This could make the initial diagnosis of concussion more difficult [24]. These individuals may also have difficulty in learning proper techniques for safe play due to characteristics such as attentional impairment, leading to an increased risk of injury [24].

Although different risk factors for concussion have been explored separately, an often overlooked area of study is the interaction between age, sex, and comorbid conditions. The identification of risk factors and how they may interact with one another after suffering a concussion can be of great value to health care providers who manage concussion symptoms and recovery. The current study examined the comorbid conditions of anxiety, depression, behavioural disorders, and learning disabilities and how they interact with age and sex to contribute to total symptoms and symptom severity after suffering a concussion.

## 2. Materials and Methods

Data were collected and stored on the Complete Concussion Management Inc. (CCMI) Concussion Database System. All information were entered into the CCMI Concussion Database System by licensed health care professionals with training in the diagnosis and management of concussion. Beginning on October 10, 2014, data were collected and entered at the time of the initial patient visit to one of the 144 CCMI-partnered clinic facilities throughout Canada. Following ethics approval from the Carleton University Research Ethics Board, deidentified patient data were extracted on November 10, 2017, to an encrypted and password-protected Excel file and electronically transferred to the principal investigator for analysis.

Inclusion criteria for the study included a diagnosis of anxiety (*n* = 609), depression (*n* = 450), a behavioural disorder (*n* = 234), or a learning disability (*n* = 284). All participants were separated into one of the six age groupings: ages 12 and under, 13–15, 16–19, 20–29, 30–39, and 40+. The younger age group clusters were chosen based on previous reports showing differences in concussion symptomology [25, 26]. A total of 1,577 participants were included in the study for the experimental data (see Table 1 for number of participants per category). Control data from 3,288 participants were extracted and separated into the same six age groupings as the experimental data (see Table 1 for number of participants per category). Participants were excluded if the time between the date of injury and their assessment was greater than 28 days; this time-frame was chosen based on the literature, as less than four weeks is still considered to be a normal recovery time in the paediatric population [27–31]. The ICD-10 criteria for “Postconcussion Syndrome” is defined as having 3 or more symptoms beyond 28 days after injury [32].

As per the postconcussion symptom severity scale (SCAT 5 [2, 33]), patients were asked to self-rate the severity of each of the 22 symptoms (headache, head pressure, neck pain, nausea, dizziness, blurred vision, balance problems, light and noise sensitivity, feeling slowed down, feeling in a fog, do not feel right, difficulty in concentrating, difficulty in remembering, fatigue, confusion, drowsiness, trouble falling asleep, more emotional, irritability, sadness, and nervous) on a 7-point Likert scale from zero to six. The symptom total was a measure of how many symptoms out of the 22 were being experienced, while the severity of each symptom was rated from 0 (not experiencing the symptom at all) to 6 (very severe). For younger patients, and those who did not understand a particular symptom, further explanation was typically given. For example: “nauseous” may be clarified by the clinician as “feeling sick to your stomach;” “fatigue” may be clarified as “feeling tired;” “irritability” may be clarified as “feeling irritated or getting frustrated with people or things easier than normal.” Clinicians were trained to ensure that all patients had a good understanding of the symptoms being asked of them. All CCMI clinicians undergo extensive concussion-specific training based on the latest scientific evidence with respect to concussion. The CCMI training course is 40 hours long, completed over 8 weeks, and covers topics including neurology, international consensus statements, pathophysiology and biomechanics of injury, pharmacological and nutritional interventions, acute concussion assessment and management, multimodal baseline and postinjury testing and interpretation/psychometric properties, postconcussion syndrome assessment, diagnosis, and management, and chronic traumatic encephalopathy. All CCMI clinicians complete this training and must also pass an examination in order to be permitted into the CCMI network. Clinicians are also required to review monthly research updates (summaries of all publications in the past month), as well as undergo biennial recertification training.

Data were collected in a self-reported manner during the process of a clinical interview. For example, participants were asked by a clinician to fill in their symptom severity scores. Questions pertaining to anxiety, depression, sleep disorders, etc., were asked during a clinical interview by way of “have you ever been diagnosed with a learning disability, ADD, or ADHD or anxiety or depression or sleep disorders or any other mental health conditions?

For the purpose of the present work, individual symptoms were not analyzed. A total symptom score (total number of symptoms experienced at the time of intake) and overall symptom severity (total score for all symptoms experienced) were calculated for each participant. Two, multivariate fix factor ANOVAs (sex and age) were carried out on the control subject data. Significant main effects and interactions were followed-up with Tukey's post hoc tests (alpha set at 0.05). Because in the control dataset, there were main effects of sex and age, we decided to generate a percent change score from the control data for the analysis of the different mental health groupings. In this way, an individual score (symptom total and severity) was divided by the mean from the respective age group and multiplied by 100. Averages were then calculated for each age grouping and sex for each of the four conditions (depression, anxiety, behavioural disorder, and learning disability). This analysis allowed us to control for the differences in symptoms between ages and sex and examine the contribution of each of the premorbid conditions. Two, multivariate fix factor ANOVAs (sex by age) were carried out on each of the 4 mental health groupings. Interactions and main effects were analyzed with Tukey's post hoc tests (alpha level set at 0.05). Statistical analyses were completed using Microsoft Excel (Microsoft Office 365 ProPlus package; Canada) and IBM SPSS software (version 25; Canada).

## 3. Results

### 3.1. Control Group (Nondiagnosed)

**Figure 1:**
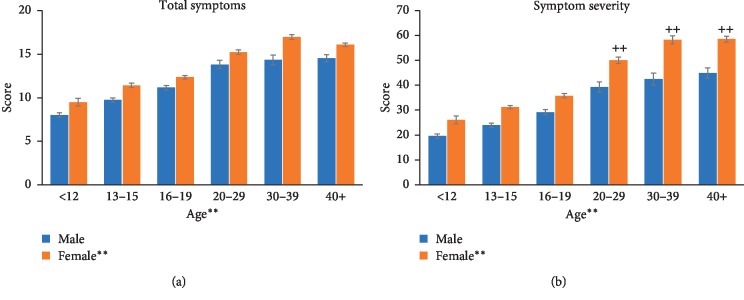
Total symptoms (a) and symptom severity (b) measured across each age group for male and female controls after suffering a concussion. (a) Total symptoms increased with age, and females experienced more symptoms than males. (b) Symptom severity also revealed higher scores with increasing age, and the difference in symptom severity score was evident between males and females in each age group but reached statistical significance in the older female groups (++, *p* < 0.01 male vs. female: 20–29, 30–39, and 40+).

The control group consisted of individuals who did not self-disclose, or have a diagnosis for, a mental health condition of depression, anxiety, a behavioural disorder, or a learning disability. Symptom total and symptom severity increased consistently with age, and females scored higher than males in each age group (Figures 1(a) and 1(b)). Total symptoms (Figure 1(a)) measured across each age group for male and female controls after suffering a concussion. A fixed-factor ANOVA did not reveal a significant interaction between age and sex (*F* (5, 3276) = 0.697), but a main effect of age (*F* (5, 3276) = 116.774, *p* < 0.001) and sex (*F* (1, 3276) = 61.416, *p* < 0.001) was found. Analysis of symptom severity (Figure 1(b)) revealed an interaction between age and sex (*F* (5, 3276) = 3.176, *p* < 0.01) and main effects of age (*F* (5, 3276) = 135.169, *p* < 0.001) and sex (*F* (1, 3276) = 134.378, *p* < 0.001). The difference in symptom severity scores was evident between males and females in each age group but was found to be significantly different in the 20 and above age groups with older females showing higher symptom severity than their age-matched males (Tukey's: *p* < 0.01 for 20–29, 30–39, and 40+; Figure 1(b)).

### 3.2. Depression

**Figure 2:**
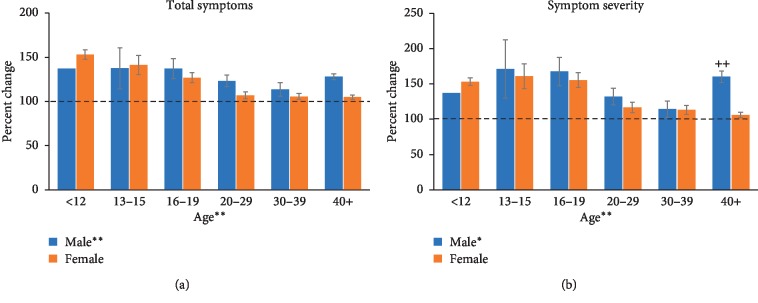
Total symptoms (a) and symptom severity (b) measured across each age group for male and female participants with a diagnosis of depression. (a) In the depression groups, total symptoms decreased with age, and males experienced more symptoms than females. (b) Symptom severity also revealed higher scores in the younger age groups, particularly evident in the 13–15 and 16–19 age groups, and the difference in symptom severity score was significant between males and females in the 40+ age group (++, *p* < 0.01 male vs. female: 40+).

In the under 12 age group, there were only 1 male and 2 females with a diagnosis of depression. The total symptoms and symptom severity values from these 3 participants are included in Figures 2(a) and 2(b) (average for the 2 females), but the data were excluded from the statistical analyses. Depression was associated with more symptoms in the 19 and under age groups, and males with depression showed more symptoms than females overall (Figures 2(a) and 2(b)). A fixed-factor ANOVA on total symptoms in the depression group did not reveal a significant interaction (*F* (4, 436) = 1.132) but did reveal main effects of age (*F* (4, 436) = 6.115, *p* < 0.001) and sex (*F* (1, 436) = 6.793, *p* < 0.01).

Symptom severity in male and female participants with depression appeared worse in the 13–15 and 16–19 age groups than the other ages. An interaction between age and sex was revealed (*F* (4, 436) = 2.641, *p* < 0.05) as well as main effects of age (*F* (4, 436) = 7.281, *p* < 0.001) and sex (*F* (1, 436) = 6.432, *p* < 0.05). Tukey's post hoc analyses revealed that the 40+ males had higher symptom severity than 40+ females (*p* < 0.01).

### 3.3. Anxiety

**Figure 3:**
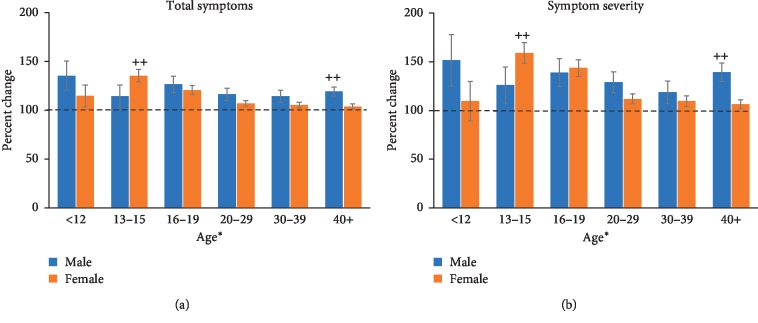
Total symptoms (a) and symptom severity (b) measured across each age group for male and female participants with a diagnosis of anxiety. (a) In the anxiety groups, total symptoms were greater in the younger groups, but there was no difference overall between males and females. Females in the 13–15 age group showed more symptoms than their age-matched males (++, *p* < 0.01) and males in the 40+ age group showed more symptoms than age-matched females (++ *p* < 0.01). (b) Symptom severity also revealed higher scores in the younger age groups, particularly evident in the 16–19 age groups, but there was no overall sex difference. The difference in symptom severity score was significant between males and females in the 13–15 with females showing greater symptom severity than males (++, *p* < 0.01) and in the 40+ age group, males showed greater symptom severity than females (++, *p* < 0.01).

A fixed-factor ANOVA on the total symptoms in male and female participants with anxiety revealed a significant interaction between age and sex (*F* (4, 436) = 2.641, *p* < 0.05). Post hoc analyses revealed that males in the 40+ age group and females in the 13–15 age group experienced significantly more symptoms than their female/male counterparts (*p* < 0.01). A main effect of age (*F* (5, 595) = 2.449, *p* < 0.05) was also found (Figure 3(a)) but no main effect of sex (*F* (1, 595) = 2.371).

A fixed-factor ANOVA on symptom severity (Figure 3(b)) revealed a significant interaction between age and sex (*F* (4, 436) = 2.374, *p* < 0.05) with the 13–15-year-old female group showing greater symptom severity than the age-matched males (*p* < 0.01). In the 40+ age group, males presented with a significantly greater symptom severity than females (*p* < 0.01). Analysis also revealed a main effect of age (*F* (5, 595) = 2.881, *p* < 0.05) but no main effect of sex (*F* (1, 595) = 1.888).

### 3.4. Behavioural Disorder

**Figure 4:**
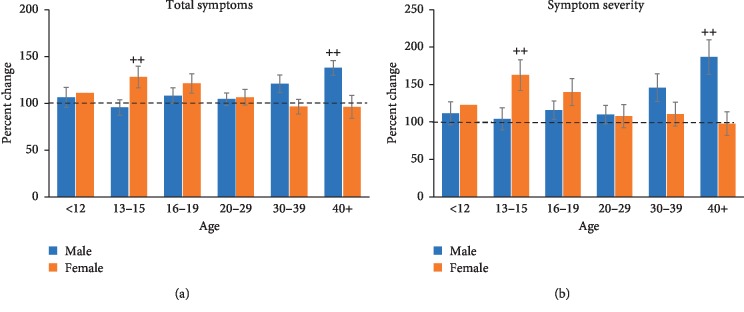
Total symptoms (a) and symptom severity (b) measured across each age group for male and female participants with a behavioural disorder. In the behavioural disorder groups, total symptoms and symptom severity showed similar, statistical trends with no main effects of age or sex. However, a significant interaction was revealed with females showing both more symptoms and greater symptom severity than males in the 13–15 age group (++, *p* < 0.01) and males showing more symptoms and more severity than females in the 40+ group (++, *p* < 0.01).

In the under 12 age group, there were only 2 females classified with a behavioural disorder. One subject experienced one symptom, and the other experienced 20 symptoms. Due to the variability, the total symptoms and symptom severity averages are included in Figures 4(a) and 4(b), but the data were excluded from the statistical analyses. Analysis revealed a significant interaction between age and sex (*F* (4, 220) = 2.673, *p* < 0.05; Figure 4(a)) but no main effects of age (*F* (5, 220) = 0.316) or sex (*F* (1, 220) = 0.226). As can be seen in Figure 4(a), females showed more symptoms than males in the 13–15 year age group (*p* < 0.01), and males showed more symptoms than females in the 40+ group (*p* < 0.01).

Analysis of symptom severity in the behavioural disorder group revealed a significant interaction between age and sex (*F* (4, 220) = 3.434, *p* < 0.05) but no main effects of age (*F* (5, 220) = 0.848) or sex (*F* (1, 220) = 0.467). Symptom severity (Figure 4(b)) was higher in the 13–15-year-old females compared with males (*p* < 0.01), and males in the 40+ group showed a higher symptoms severity than females (*p* < 0.01).

### 3.5. Learning Disability

**Figure 5:**
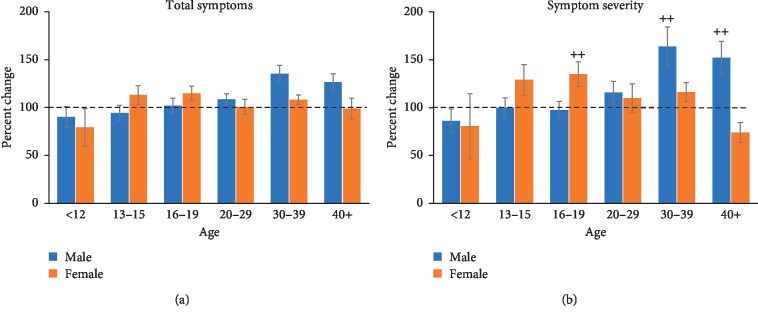
Total symptoms (a) and symptom severity (b) measured across each age group for male and female participants with a learning disability. In the learning disability groups, total symptoms were not affected by age or sex and did not seem to differ from the control values. Measurement of symptom severity showed similar, statistical trends with no main effects of age or sex. However, a significant interaction was revealed with females showing greater symptom severity than males in the 13–15 and 16–19 age group (++, *p* < 0.01) and males showing more severe symptoms than females in 30–39 and 40+ age groups (++, *p* < 0.01).

Fixed-factor analysis on total symptoms in the learning disability group revealed no main effect of age (*F* (5, 271) = 1.597), no main effect of sex (*F* (1, 271) = 1.050), and no significant interaction between age and sex (*F* (5, 271) = 1.636; Figure 5(a)).

Analysis of symptom severity data (Figure 5(b)) revealed a significant interaction between age and sex (*F* (5, 271) = 3.374, *p* < 0.01). Tukey's post hoc analysis revealed greater symptom severity in the 16–19-year-old females compared with the age-matched males (*p* < 0.01), and symptom severity was greater in the 30–30 and 40+ male age groups compared with females (*p* < 0.01 for both comparisons). There were no main effects of age (*F* (5, 271) = 1.562) or sex (*F* (1, 271) = 1.386).

## 4. Discussion/Conclusions

The goal of this study was to analyze age, sex, and four mental health conditions (depression, anxiety, behavioural disorders, and learning disabilities) as risk factors for symptom total and symptom severity presentation in concussed participants. Of particular interest was whether these diagnoses exacerbated symptoms above and beyond those reported in nondiagnosed (control) concussed participants. Symptoms were assessed using the Postconcussion Symptom Scale (PCSS), which contains a list of 22 common concussion symptoms that patients must rank on a scale from 0 (not experiencing a given symptom at all), to 6 (the symptom is “severe”). Since some of these symptoms are not specific to concussion and could be caused by other etiologies, patients are instructed to rate only those symptoms that started after suffering their concussion [14]. However, it can be difficult to differentiate symptoms based on their individual causes, especially with self-report studies. The increased percent change that we see in the patients with a mental health condition could be because they are reporting symptoms that were present before the concussion occurred, as well as symptoms that are exacerbated by the concussion. Almost half of patients with persistent postconcussion symptoms suffer from depression and anxiety [34]. The question of causality is unclear, i.e., whether pre-existing mental health conditions lead to greater symptomology or concussion symptomology lead to a worsening of the pre-existing mental health conditions [17, 35–38].

In the concussed, control groups, total symptoms and symptom severity increased with age, with females showing a greater number of symptoms and significantly greater symptom severity than males. The difference between females and males seemed to be more prominent in the older ages with symptom severity reaching significance in the 20–29, 30–39, and 40+ age categories. The demographic data presented in Table 1 show that males reported more concussive injuries than females across all age categories with more pronounced differences in the 20–29 and 30–39 age groups. Based on this, it does not appear that the number of concussions accounts for the greater symptom severity in females. There are various reasons as to why females experience more symptoms and greater severity of symptoms after suffering a concussion compared with their male counterparts. Biomechanical differences seem to contribute to symptom status due to disparities in the neck musculature and head/neck stability in females making them more susceptible to injury [6]. Males also have greater cortical neuronal densities, while females have a greater number of neuropils [11]. Females also have a greater blood flow rate and exhibit a greater basal rate of glucose metabolism. The typical decrease in cerebral blood flow along with the increased glycemic demands caused by a brain injury may interact with the already increased metabolic demands in females, leading to greater impairment in females [11].

Hormonal factors may also play a role in the differential response to injury seen between males and females. Studies have shown that estrogen serves a protective factor in males, but it exacerbates the injury in females [11]. The menstrual cycle is also a predictor of outcomes after a concussion. Women who get injured during the last two weeks of the cycle (luteal phase) have worse symptoms than women injured during the follicular phase [11]. There is also evidence that females report more symptoms and a greater severity of symptoms than males after suffering a concussion because it is more socially acceptable for women to admit vulnerability than it is for men [6]. However, Broshek et al. [11] reported that females do in fact experience more severe postconcussion symptoms than males and that poorer outcomes are not simply due to cultural-based sex differences in symptom reporting.

Symptom total and symptom severity increased with age in the control groups. This finding is consistent with the majority of the literature as it is generally reported that increased age is associated with worse outcomes following a concussion [12]. One explanation for this is that neuroplasticity may decline with age. Neuroplasticity can be defined as neurobiological processes that result in the stability or compensation for disease-related changes [39]. This means that older individuals will have a harder time recovering from injury and may experience an injury more severely due to a lowered capacity for compensation. Older participants may also be at a higher risk for progressive cognitive decline after suffering a concussion because of pre-existing comorbidities [12]. This also makes the assessment of concussion challenging in the older population as the presence of pre-existing conditions can make it difficult to isolate the effect of a concussion [40].

For those who did have a mental health condition of either depression, anxiety, a behavioural disorder, or a learning disability, concussion symptom and severity were exacerbated across most of the age groups for each condition. A diagnosis of anxiety or depression exacerbated the symptoms in the 12 and under, 13–15, and 16–19 age groups the most. Existing literature suggests that younger athletes take longer to recover from concussion than older athletes, especially those in high school [14]. The high school years are those from age 15–18 which encompasses the 13–15 and 16–19 age groups in our study. Perhaps we see an exacerbated symptom presentation in this age group of individuals with depression and anxiety because these individuals are already at an increased risk of prolonged symptoms.

Females with a diagnosis of anxiety in the 13–15 age group showed a significantly greater number of symptoms compared with their male counterparts. A similar pattern was observed in 13–15-year-old females with a diagnosis of a behavioural disorder. Symptom severity was also significantly greater in females with a behavioural disorder than in males. Female sex has been shown to be associated with prolonged recovery from concussion, as well as an increased severity of symptoms experienced [14]. Symptom total and symptom severity were exacerbated in the 19 and under females more than males for all four conditions, with the exception of the youngest age group (12 and under). Guerriero et al. [14] suggested that because adolescence itself is a period of heightened vulnerability given the maturation of affective and regulatory systems, all adolescents, regardless of mental health status, might demonstrate a range of affective feelings that could raise their postconcussion symptom scores after injury. On top of that, the maturation of these emotional and regulatory systems is what likely leads to the exacerbation of somatic complaints that can be seen throughout adolescence [14].

Males in the 40+ age groups scored noticeably higher for both symptom total and symptom severity in all of the mental health conditions. While the literature is dominated by studies reporting that females experience greater symptoms and symptom severity than males [14], most of those studies are carried out with younger cohorts such as high school- and university-aged participants. There are a few reasons as to why we might only see this drastic percent change in 40+ males, while the females hovered around control values. First, it may be due to the potential under-reporting of symptoms and severity by males in the control group. If the males in the experimental group were more honest about their symptomology than the control group, this could contribute to the jump in symptom total and severity between the two groups. However, there is no evidence that males with a mental health condition are any more honest about their symptoms than those in the control group. Second, the female control scores are already much higher than the males, which is why we might see such a noticeable change in the males in the experimental groups. Finally, if we accept the results for what they are, perhaps males over 40 years old with a mental health condition are simply at a greater risk of increased symptoms and severity of those symptoms.

Most research on comorbid conditions in relation to concussion outcome has focused on depression and anxiety [14, 35, 37, 41–44]. More and more work has shown a link between behavioural disorders (such as ADHD) and learning disabilities as contributing factors to concussion symptom and severity presentation [45–48]. As an example, participants with ADHD were significantly more disabled after suffering a concussion than were control participants without ADHD, even when controlling for age and sex [49–53]. They concluded that individuals who sustain a concussion in the presence of a comorbid diagnosis of ADHD are more likely to experience greater symptoms and greater symptom severity.

There is an increased need for mental health screening in athletes to identify those at risk for postconcussion syndrome. The early identification of at-risk individuals allows for health care professionals and family members too have more realistic expectations regarding the duration of symptoms and symptom management [54]. Further research would be beneficial in addressing the potential benefit of mental health screening and its impact on concussion treatment, as there is limited awareness on this topic. Evaluating baseline psychological function as well as family history of mental health conditions could assist in identifying individuals at risk of prolonged symptoms or increased severity of symptoms. The future treatment of concussion symptoms should take on a multidisciplinary approach. Mental health support should also be an essential component of any interdisciplinary care team in concussion clinics.

The consideration of risk factors in concussion management and how these risk factors affect symptom outcome after suffering a concussion are very important. The purpose of this study was to look at the interaction between age, sex, and comorbid mental health conditions and how these factors affect symptom presentation after suffering a concussion. For those without a mental health condition, symptom total and symptom severity increased with age with females scoring higher than males across all ages. A diagnosis of anxiety or depression exacerbated symptoms in the 19 and under age groups as well as males 40 years and older. A diagnosis of a behavioural disorder or a learning disability exacerbated symptoms in the 13–19-year-old females and males 30 years and older. The association between comorbid conditions and duration of concussion symptoms are important to consider when implementing concussion management strategies. The potential for sex differences and other unidentified variables that affect concussion severity and recovery should also be considered when applying individualized treatment. Medical care as well as return-to-play decisions should be based on careful analysis of each individual's history and needs rather than a one-size-fits-all criteria. Future research is needed to further understand how to better treat individuals that present with anxiety, depression, a behavioural disorder or a learning disability as the current literature is limited. This includes understanding the mechanism of interaction between preinjury mental health conditions and postconcussion symptoms. The benefit of mental-health screening for at-risk individuals should also be considered when looking at ways to enhance postconcussion recovery.

## Figures and Tables

**Table 1:** Number of participants (*n*) for each condition, sex, and age group. Included in this table are the average number of concussions (tc: total concussions) reported for each condition, sex and age group. Males in the age range of 13–15 with a diagnosis of depression showed a higher average of concussions (2*x*) than controls. Males in the 30–39 and 40+ age groups with a learning disability showed a higher average number of concussions (2*x*) than the control group.

Group	12 and under		13–15		16–19		20–29		30–39		40+	
Control	*n*	tc	*n*	tc	*n*	tc	*n*	tc	*n*	tc	*n*	tc
Male	351	*1.75*	507	*1.88*	425	*2.06*	148	*2.67*	102	*2.31*	156	*1.65*
Female	162	*1.51*	438	*1.67*	339	*1.89*	213	*1.95*	144	*1.70*	303	*1.34*
Depression												
Male	1	*1.0*	10	***3.6***	20	*3.05*	28	*2.5*	24	*2.87*	35	*2.26*
Female	2	*1.5*	18	*2.11*	55	*2.4*	64	*2.56*	61	*2.0*	131	*1.78*
Anxiety												
Male	23	*1.39*	31	*2.06*	35	*3.09*	38	*2.13*	25	*2.24*	41	*2.54*
Female	16	*1.44*	54	*1.91*	87	*2.32*	84	*2.25*	68	*2.13*	106	*1.71*
Learning disability												
Males	26	*1.54*	58	*2.03*	39	*2.72*	29	*2.34*	8	***4.5***	10	***3.4***
Females	8	*2.0*	28	*1.61*	34	*2.24*	18	*2.28*	15	*1.6*	10	*2*
Behavioural disorder												
Males	32	*1.59*	40	*2.18*	40	*2.3*	24	*2.46*	16	*2.69*	6	*1.83*
Females	2	*2.0*	15	*2.53*	21	*2.29*	14	*2.57*	12	*2.0*	11	*2.55*

## Data Availability

The CCMI data used to support the findings of this study were supplied by Dr. Cameron Marshall under license and so cannot be made freely available. Requests for access to these data should be made to Dr. Cameron Marshall, Complete Concussion Management, Inc. Address: 2655 Bristol Cir, Oakville ON L6H 7W1.
